# Falco: high-speed FastQC emulation for quality control of sequencing data

**DOI:** 10.12688/f1000research.21142.2

**Published:** 2021-01-27

**Authors:** Guilherme de Sena Brandine, Andrew D. Smith

**Affiliations:** 1 Department of Quantitative and Computational Biology, University of Southern California, Los Angeles, California, 90089, USA

**Keywords:** FastQC, high-throughput sequencing, quality control

## Abstract

Quality control is an essential first step in sequencing data analysis, and software tools for quality control are deeply entrenched in standard pipelines at most sequencing centers. Although the associated computations are straightforward, in many settings the total computing effort required for quality control is appreciable and warrants optimization. We present Falco, an emulation of the popular FastQC tool that runs on average three times faster while generating equivalent results. Compared to FastQC, Falco also requires less memory to run and provides more flexible visualization of HTML reports.

## Introduction

High-throughput sequencing is routinely used to profile copy number variations in cancers ^1^, assemble genomes of microbial organisms ^2, 3^, quantify gene expression ^4^, identify cell populations from single-cell transcriptomes in a variety of tissues ^5^, and track epigenetic changes in developing organisms and diseases ^6^, among numerous other applications. New sequencing protocols are constantly being introduced ^7, 8^, and as the cost of sequencing per base decreases, sequencing data is growing in abundance, dataset size, and read length ^9^.

Quality control (QC) is often the first step in high-throughput sequencing data analysis pipelines. The QC step measures a set of statistics in a file of sequenced reads to assess if its content matches the experiment expectations and if the data is suitable for downstream analysis. Common QC tests include counting relative frequency of nucleotides in each position of a set of reads to detect potential deviations from expected frequencies, summarizing the distribution of Phred ^10^ quality scores to identify base positions with globally low quality (suggesting degeneration in the sequencing process), and measuring the frequency of sequencing adapters and contaminants that are not expected to be biological DNA from the sample.

Data that passes specific QC tests then undergoes downstream analysis steps, which may include adapter trimming, filtering contaminants and low-quality reads, and mapping the resulting reads to a reference genome or transcriptome. With the exception of sequence assembly applications, read mapping should be the most computationally expensive step early in analysis pipelines. In comparison, the time and computation required for QC should be negligible. However, the efficiency of mapping algorithms has improved substantially over the past decade, while software for QC has received far less attention. As a consequence, the computation required for QC is appreciable, and can no longer be ignored when considering the total cost of sequencing.

The most commonly used tool for quality control of sequencing data is FastQC ^11^, which, since its release, has incorporated a wide range of QC tests covering multiple use cases. Its analysis reports have become the standard for several QC tools, and automated analysis pipelines often rely on its result as a criterion to proceed with downstream steps or, alternatively, to filter, trim, or ultimately discard the data ^12, 13^. FastQC reports ten analysis modules that summarize the content of a sequencing file ( Table 1). An input file may pass or fail the tests run in each module, and high-quality sequencing data from most protocols is expected to pass all tests.

In FastQC’s implementation, each module computation is executed sequentially after an input sequence is read. This design allows new modules to be incorporated easily, but it implies that the time required to process each read is the sum of the processing times for each module. If multiple modules compute similar measurements, such as nucleotide content or Phred quality scores, the same calculation will be performed multiple times, causing the total analysis run time to increase.

Several QC software tools have been introduced since FastQC, many focusing on speed improvements, more flexible module visualization, incorporation of paired-end reads, and filtering sequences that failed QC tests. Despite proposing different alternatives to calculate and present QC results, the modules available in these tools are largely similar to FastQC’s ( Table 1).

**Table 1.** Comparison of analysis modules provided by fastp and HTQC, two commonly used QC software tools.

FastQC module	fastp	HTQC
Per base sequence quality	No	Yes
Per base N content	Yes	Yes
Per tile sequence quality	No	Yes
Per sequence quality scores	No	Yes
Per sequence GC content	No	No
Sequence length distribution	No	Yes
Sequence duplication levels	Yes	No
Overrepresented sequences	Yes	No
Adapter content	No	No
Kmer content	Yes	No

At the same time, FastQC’s analysis results are already part of many standard initial analysis pipelines. If a new QC software tool is incorporated in these pipelines, it is desirable that its results, and its output formats, remain consistent with those of FastQC.

To address potential speed limitations in FastQC’s implementation while retaining its behavior, we developed *FastQC Alternative Code* ( Falco) ^14^, an emulation of the FastQC software tool. We show that Falco generates the same results as FastQC across a wide variety of datasets of different read lengths, sizes, file formats, and library preparation protocols at significantly shorter running times and using less memory. While the text outputs are comparable to FastQC, Falco also provides more flexible interaction with graphical plots in its HTML report using the same visualization standards set by FastQC.

## Methods

### Implementation choices

Falco ^14^ is an Open Source C++ implementation of the FastQC software tool built for UNIX-based operating systems. We designed to faithfully emulate FastQC’s calculations, results and text reports. The goal of Falco is to minimize the effort required to replace the command-line behavior of FastQC in the context of larger automated analysis pipelines. We use the same set of command-line arguments, configuration file names, and input file formats as FastQC. We also produce the same plain text format output, and the same report structure, allowing users to take advantage of improved speed without adjusting to different program behaviors. Falco is intended to be used in a command-line environment. Unlike FastQC, Falco cannot be run through a graphical user interface.

There are major differences between the implementations of Falco and FastQC. While FastQC’s code emphasizes modularity, which allows new QC metrics to be added easily and uniformly, Falco’s design centralizes the function to read sequences from the input file and collects the minimum data necessary to subsequently create all modules after file processing. To ensure consistency with FastQC, we wrote each module’s source code based on FastQC’s implementation, adapting the portions that relate to sequence processing and maintaining the postprocessing functions that define how the collected data is used to generate summaries and reports.

### Operation

Compilation of Falco requires a GNU GCC compiler version 5.0.0 (July 16, 2015; full support for the C++11 standard) or greater. Once compiled, Falco can be run on uncompressed files (FASTQ and SAM) without any additional dependencies. In order to process files in gzip compressed FASTQ and BAM formats, Falco must be compiled with the ZLib ^15^ and HTSLib ^16^ libraries, respectively. The full documentation on how to compile, install dependencies, and run the program is available in the README file in the Falco repository.

## Use cases

Like FastQC, Falco ^14^ can be applied to any file of sequenced reads in the formats accepted by FastQC. The only required command-line argument is the path to the input file. Also like FastQC, a wide range of options can be provided if users only require a given subset of its analysis modules or outputs. The letters and symbols used for command-line arguments were chosen to maintain consistency with FastQC’s options. As mentioned above, this choice is to facilitate integration with larger pipelines that already employ FastQC and depend on its behaviors.

Falco can be run on a FASTQ format file named example.fq with the following simple command:

$ falco example.fq

This will generate three files:

1. fastqc_data.txt: The complete numerical values generated in each module’s individual analysis.
2. fastqc_report.html: A visual page display of the text report’s data and plots generated in modules.
3. summary.txt: A short summary indicating whether the input file passed or failed each module, and whether any warnings were raised.

Default configuration files are contained in a Configuration directory that is included with the program, but Falco also allows users to manually define the thresholds to pass or fail each module, the list of adapters to search for in reads, and the list of contaminants to compare with overrepresented sequences by using configuration files in the same format used by FastQC.

### System requirements

Falco requires little memory and disk space to run, and there are no constraints on the minimum or maximum FASTQ input size or number of reads. Reads are analyzed sequentially, with one read stored in memory at a time, so the amount of memory necessary to run depends on the largest read length in a dataset, but not on the size of the input file. For instance, processing a short-read sample, with reads of length at most 1000 bases, requires 100 MB of available RAM, whereas processing a long-read sample containing at least one read with 1 million bases require 500 MB of RAM. The total disk space necessary to store the three output files generated by Falco is no more than 1 MB.

## Results

### Falco matches FastQC’s output

We compared the output of Falco ^14^ to its FastQC counterpart using 11 datasets ( Table 2). The tests consist of Illumina files originating from a range of different library preparation protocols for DNA, RNA, and epigenetic experiments, as well as reads from the nanopore ^17^ technology. For simplicity, Illumina paired-end datasets were only tested on the first read end.

**Table 2.** Datasets used for comparison with FastQC’s output and run time speed benchmarking between QC tools.

test	accession	reference	file size (FASTQ)	reads	length (bp)	protocol
1	SRR10124060	unpublished	7.3GB	25,172,255	130	RNA-Seq
2	SRR10143153	unpublished	11.0GB	15,949,900	150	miRNA-Seq
3	SRR3897196	23	4.2GB	15,570,348	100	BS-Seq
4	SRR9624732	24	1.6GB	18,807,797	150	ChIP-Seq
5	SRR1853178	25	130.0GB	510,210,716	60	Drop-Seq
6	SRR6387347	26	20.0GB	305,434,830	100	10x genomics
7	SRR6954584	5	56.0GB	152,853,013	150	Microwell-Seq
8	SRR891268	27	46.0GB	192,904,649	50	ATAC-Seq
9	SRR9878537	unpublished	38.0MB	3,284	64,000	Nanopore
10	wgs-FAB49164	19	8.4GB	746,333	180,000	Nanopore
11	SRR6059706	unpublished	1.4GB	892,313	150,000	Nanopore

FASTQ files available in the Sequence Read Archive (SRA) ^18^ were downloaded using the fastq-dump command from the SRA toolkit. We used the following flags when running fastq-dump: -skip-technical, -readids, -read-filter pass, -dumpbase, -split-3 and -clip. One dataset was downloaded from the Whole Human Genome Sequencing Project ^19^.

We directly compared the text summary for each output of Falco to FastQC’s output summary files, obtaining the same outputs (pass, warning, or fail) for all tested criteria in all datasets.

To assess if Falco’s output is consistent with FastQC’s format, we used the fastqcr ^20^ R package version 0.1.2 and MultiQC ^12^ version 1.9. Both tools can successfully parse the text reports generated by Falco for the tested files. Differences in the fastqc_data.txt files between the two programs result from choices for numerical precision output, or as a result of Falco calculating certain averages based on more of the data within each file.

### Falco is faster than popular QC tools

Some alternative software tools exist for quality control of sequencing data, and users may opt for them due to their efficiency in cases where not all FastQC analysis modules are necessary. Among these, fastp ^21^ has gained popularity for its speed and versatile set of options for trimming. fastp has demonstrated superior runtime to FastQC even when generating FASTQ format output files corrected by trimming adapters and filtering (which requires both input and output). HTQC ^22^ is another tool that was developed with the intent to both improve speed performance and incorporate trimming functions after quality control. The two programs were used as benchmarks to compare Falco with.

Although most fastp modules are both calculated and displayed equivalently to FastQC, one major difference between these tools is how overrepresented sequences are estimated. While fastp counts the sequences at every *P* reads (which users may specify), FastQC stores the first 100,000 reads encountered for the first time, and subsequently checks if the following sequences match any of the stored candidates. This choice of implementation causes fastp’s runtime to greatly differ when overrepresentation is enabled. Conversely, FastQC’s runtime does not seem to be affected by disabling the overrepresented sequences module. For a comprehensive comparison between programs, we have measured the run times for our test datasets both with and without the overrepresented sequences module enabled. Programs were compared both in compressed (gzip FASTQ) and uncompressed (plain FASTQ) file formats.

Files used to assess Falco’s output comparison to FastQC ( Table 2) were also used for speed and memory comparison. Tests were executed in an Intel Xeon CPU E5-2640 v3 2.60GHz processor with a CentOS Linux 7 operating system. All file I/O was done using local disk to reduce variability in execution runtime. Both fastp and FastQC were instructed to run using a single thread.

FastQC version 0.11.8 was run with default parameters and the configuration limits, adapters and contaminants provided with the software. fastp version 0.20.0 was run with the -A, -G, -Q and -L flags to disable adapter trimming, poly-G trimming, quality filtering and length filtering, thus requiring the program to only perform QC tests without generating a new FASTQ file. When testing for overrepresented sequences, we set the -p flag to enable this module, and set the frequency of counts to the program’s default value of *P* = 20. We ran the ht-stat program on the tested files using the -S flag for single-ended reads. HTQC was not tested on gzip FASTQ files as this file format is not accepted by the program. We used the GNU time command to measure the total running times for each program, using the total elapsed wall time as measurement. The benchmarking results ( Table 3 and Table 4) show that Falco performs faster than fastp and FastQC in all datasets, with an average 3 times faster runtime than FastQC, both with the overrepresented sequences module on and off. Despite HTQC failing to process most test datasets due to unaccepted header formats, the two tests that ran to completion demonstrate that Falco’s analysis times are also significantly smaller in comparison.

**Table 3.** Real run times for Falco, fastp and FastQC on uncompressed FASTQ format, with the overrepresented sequences module on and off. Asterisks (*) indicate tests in which tools did not run to completion.

test	Falco	fastp	FastQC	Falco	fastp	FastQC	HTQC
	overrep off	overrep off	overrep off	overrep on	overrep on	overrep on	
1	48s	1m54s	3m30s	55s	5m57s	3m23s	12m09s
2	36s	1m20s	2m08s	37s	4m32s	2m10s	*
3	27s	1m04s	1m25s	30s	2m16s	1m24s	*
4	44s	1m48s	2m40s	51s	3m37s	2m38s	*
5	15m49s	35m14s	41m27s	15m58s	44m30s	37m43s	*
6	7m59s	18m42s	22m59s	8m33s	42m50s	22m53s	134m42s
7	6m05	13m50s	19m42s	6m49s	41m55s	19m52s	*
8	5m12s	11m47s	13m59s	5m20s	15m25s	14m08s	*
9	1s	1s	6s	1s	0m26s	6s	*
10	1m37s	1m50s	3m11s	1m32s	4m37s	3m16s	*
11	13s	24s	43s	13s	1m07s	46s	*

**Table 4.** Real run times for Falco, fastp and FastQC on gzip compressed FASTQ format.

test	Falco	fastp	FastQC	Falco	fastp	FastQC
	overrep off	overrep off	overrep off	overrep on	overrep on	overrep on
1	1m19s	2m19s	3m49s	1m25s	6m23s	3m50s
2	45s	1m31s	2m21s	51s	5m23s	2m24s
3	33s	1m10s	1m35s	35s	2m26s	1m36s
4	1m01s	2m06s	3m01s	1m03s	3m59s	3m00s
5	16m05s	42m40s	44m57s	18m17s	53m09s	44m59s
6	12m26s	23m18s	26m39s	12m29s	47m32s	26m38s
7	8m40s	17m34s	22m31s	8m34s	44m41s	22m31s
8	7m08s	14m37s	16m06s	6m31s	18m19s	16m11s
9	2s	1s	7s	1s	27s	7s
10	2m23s	2m32s	4m01s	2m34s	5m22s	4m09s
11	22s	31s	48s	23s	1m14s	51s

The memory required to run Falco differs between short-read samples (tests 1-8; Table 2) and long-read samples (tests 9-11). All programs demonstrated similar behavior in memory usage, with all short-read samples having similar memory requirements, and test 10 requiring the most memory (as it contains the longest read). The total memory usage was also measured by GNU time command. For Falco, short-read samples required 92 MB of RAM, whereas long-read samples used at most 342 MB of RAM. In short-read samples, FastQC and fastp used 319 MB and 568 MB of RAM, respectively. In long-read samples, FastQC and fastp used at most 4.88 GB and 1.28 GB of RAM, respectively. This comparison suggests that Falco’s memory requirement is also the lowest across all tests.

### Falco allows dynamic visualization of results

**Figure 1.**
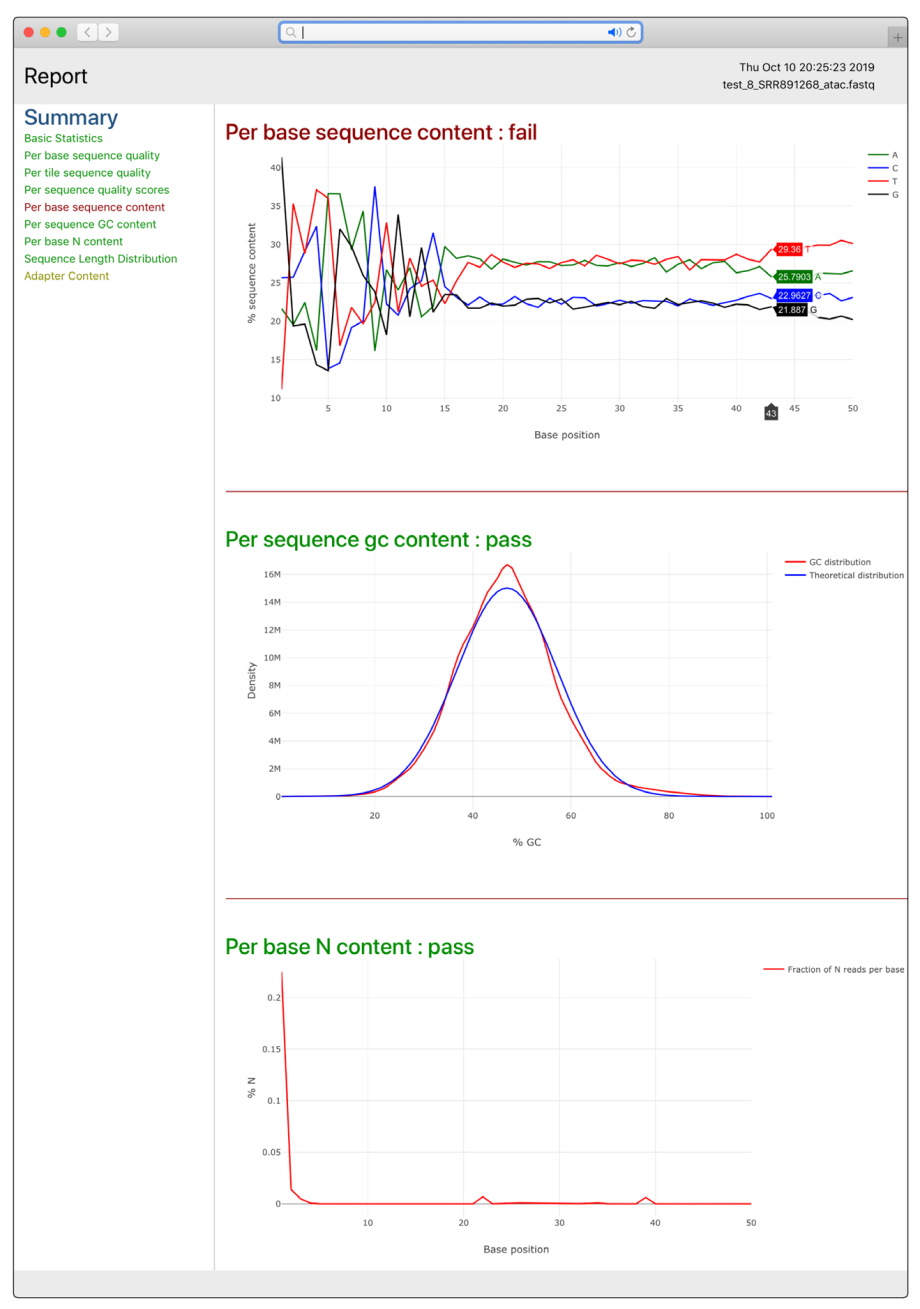
Sample HTML report for test 8 (accession SRR891268). Layout and plots are based on FastQC’s HTML report.

Despite FastQC’s clarity in its HTML reports, graphs are displayed as static images and have limited visualization flexibility, such as tile heatmaps not displaying raw deviations from average Phred scores in base positions, or raw values in line plots not being visible. We have opted to display Falco’s analysis results using the Plotly JavaScript library ^28^, which allows interactive changes of axis labels, hovering on data points to visualize raw values, and screenshots from specific positions on the plot ( Figure 1). This choice of presentation provides greater options to explore and interpret QC results while maintaining the visualization standards set by FastQC.

## Conclusions

Falco ^14^ is a faster alternative to calculate the wide range of QC metrics reported by FastQC. It is entirely based on emulating the analysis modules FastQC provides while running faster than popular QC tools and generating dynamic visual summaries of analysis results. Falco’s text output provides the same information generated by FastQC, so tools that parse this file for custom visualization and downstream analysis can seamlessly incorporate Falco into their pipeline.

## Data availability

Datasets used to compare Falco and FastQC are shown in Table 2. Guidance for how to accept accession wgs-FAB49164 is available from the Benchmark directory of the Falco GitHub page.

## Software availability

**Source code for Falco available at:** https://github.com/smithlabcode/falco.

Users may report errors, bugs, installation problems, and improvement suggestions in the same page provided to download the source code under the “issues” section.

The scripts used to download files and reproduce the benchmarking steps described are also available in the same repository within the “benchmark” directory.

**Archived source code at time of publication:** http://doi.org/10.5281/zenodo.4429381 ^14^.

**License:** GNU General Public License version 3.0.

## References

[1] Alkan C Kidd JM Marques-Bonet T: Personalized copy number and segmental duplication maps using next-generation sequencing. *Nature Genetics.* 2009;41(10):1061–1068. 10.1038/ng.437 19718026 PMC2875196

[2] Loman NJ Quick J Simpson JT: A complete bacterial genome assembled *de novo* using only nanopore sequencing data. *Nature Methods.* 2015;12(8):733–738. 10.1038/nmeth.3444 26076426

[3] Masella AP Bartram AK Truszkowski JM: PANDAseq: paired-end assembler for illumina sequences. *BMC Bioinformatics.* 2012;13(1):31. 10.1186/1471-2105-13-31 22333067 PMC3471323

[4] Ozsolak F Milos PM: RNA sequencing: advances, challenges and opportunities. *Nature Reviews Genetics.* 2011;12(2):87–98. 10.1038/nrg2934 21191423 PMC3031867

[5] Han X Wang R Zhou Y: Mapping the mouse cell atlas by Microwell-Seq. *Cell.* 2018;172(5):1091–1107.e17. 10.1016/j.cell.2018.02.001 29474909

[6] Buenrostro JD Wu B Chang HY: ATAC-seq: A method for assaying chromatin accessibility genome-wide. *Current Protocols in Molecular Biology.* 2015;109(1):21.29.1–9. 10.1002/0471142727.mb2129s109 25559105 PMC4374986

[7] Datlinger P Rendeiro AF Schmidl C: Pooled CRISPR screening with single-cell transcriptome readout. *Nature Methods.* 2017;14(3):297–301. 10.1038/nmeth.4177 28099430 PMC5334791

[8] Spanjaard B Hu B Mitic N: Simultaneous lineage tracing and cell-type identification using CRISPR–Cas9-induced genetic scars. *Nature Biotechnology.* 2018;36(5):469–473. 10.1038/nbt.4124 29644996 PMC5942543

[9] Svensson V Vento-Tormo R Teichmann SA: Exponential scaling of single-cell RNA-seq in the past decade. *Nature Protocols.* 2018;13(4):599–604. 10.1038/nprot.2017.149 29494575

[10] Ewing B Hillier L Wendl MC: Base-calling of automated sequencer traces using Phred. *Genome Res.* 1998;8(3):175–185. 10.1101/gr.8.3.175 9521921

[11] Andrews S: FastQC: a quality control tool for high throughput sequence data.2010 Reference Source

[12] Ewels P Magnusson M Lundin S: MultiQC: summarize analysis results for multiple tools and samples in a single report. *Bioinformatics.* 2016;32(19):3047–3048. 10.1093/bioinformatics/btw354 27312411 PMC5039924

[13] Brown J Pirrung M McCue LA: FQC Dashboard: integrates FastQC results into a web-based, interactive, and extensible FASTQ quality control tool. *Bioinformatics.* 2017;33(19):3137–3139. 10.1093/bioinformatics/btx373 28605449 PMC5870778

[14] De Sena Brandine G Smith A: smithlabcode/falco: 0.2.4 - 2019/10/28.2019 10.5281/zenodo.4429381

[15] Deutsch P Gailly JL: Zlib compressed data format specification version 3.3.1996 10.17487/RFC1950

[16] Li H Handsaker B Wysoker A: The Sequence Alignment/Map format and SAMtools. *Bioinformatics.* 2009;25(16):2078–2079. 10.1093/bioinformatics/btp352 19505943 PMC2723002

[17] Jain M Olsen HE Paten B: The Oxford Nanopore MinION: delivery of nanopore sequencing to the genomics community. *Genome Biology.* 2016;17(1):239. 10.1186/s13059-016-1103-0 27887629 PMC5124260

[18] Leinonen R Sugawara H Shumway M: The sequence read archive. *Nucleic Acids Res.* 2010;39(Database issue):D19–D21. 10.1093/nar/gkq1019 21062823 PMC3013647

[19] Jain M Koren S Miga KH: Nanopore sequencing and assembly of a human genome with ultra-long reads. *Nature Biotechnology.* 2018;36(4):338–345. 10.1038/nbt.4060 29431738 PMC5889714

[20] Kassambara A: fastqcr: Quality control of sequencing data. R package version 0.1.2.2019 Reference Source

[21] Chen S Zhou Y Chen Y: fastp: an ultra-fast all-in-one FASTQ preprocessor. *Bioinformatics.* 2018;34(17):i884–i890. 10.1093/bioinformatics/bty560 30423086 PMC6129281

[22] Yang X Liu D Liu F: HTQC: a fast quality control toolkit for Illumina sequencing data. *BMC Bioinformatics.* 2013;14(1):33. 10.1186/1471-2105-14-33 23363224 PMC3571943

[23] Decato BE Lopez-Tello J Sferruzzi-Perri AN: DNA methylation divergence and tissue specialization in the developing mouse placenta. *Molecular Biology and Evolution.* 2017;34(7):1702–1712. 10.1093/molbev/msx112 28379409 PMC6440273

[24] Yang J Zhang L Jiang Z: TCF12 promotes the tumorigenesis and metastasis of hepatocellular carcinoma via upregulation of CXCR4 expression. *Theranostics.* 2019;9(20):5810–5827. 10.7150/thno.34973 31534521 PMC6735379

[25] Macosko EZ Basu A Satija R: Highly parallel genome-wide expression profiling of individual cells using nanoliter droplets. *Cell.* 2015;161(5):1202–1214. 10.1016/j.cell.2015.05.002 26000488 PMC4481139

[26] Nusse YM Savage AK Marangoni P: Parasitic helminths induce fetal-like reversion in the intestinal stem cell niche. *Nature.* 2018;559(7712):109–113. 10.1038/s41586-018-0257-1 29950724 PMC6042247

[27] Buenrostro JD Giresi PG Zaba LC: Transposition of native chromatin for fast and sensitive epigenomic profiling of open chromatin, DNA-binding proteins and nucleosome position. *Nature Methods.* 2013;10(12):1213–1221. 10.1038/nmeth.2688 24097267 PMC3959825

[28] Sievert C Parmer C Hocking T: plotly: Create interactive web graphics via ‘plotly. js’.2017 Reference Source

