# Supplementary material for: Falco: high-speed FastQC emulation for quality control of sequencing data
Source: F1000Res. 2021 Jan 27;8:1874. Originally published 2019 Nov 7. [Version 2] doi: 10.12688/f1000research.21142.2 (PMC7845152; doi:10.12688/f1000research.21142.2)
Supplement: Peer review history

# Peer review: Reviewer response for version 2

**DOI:** 10.5256/f1000research.46637.r78323

**Published:** 2021-01-28

**Authors:** R. Henrik Nilsson

**Affiliations:** 1 Gothenburg Global Biodiversity Centre, University of Gothenburg, Gothenburg, Sweden

I am happy with the revised manuscript*. It packs quite some punch.

 But I attach some few discretionary, cosmetic suggestions in the below.

**Methods:**

- ”is Open Source” – shouldn’t this be ”is open source” here? Not a proper noun and not a capitonym, after all?
- “calculations, results and text reports.” > “calculations, results, and text reports.”

**Results:**

- “configuration limits, adapters and contaminants” > “configuration limits, adapters, and contaminants”.
- “trimming, quality filtering and length filtering” > “trimming, quality filtering, and length filtering”.
- “Real run times for Falco, fastp and FastQC” > ““Real run times for Falco, fastp, and FastQC””.

Are the conclusions about the tool and its performance adequately supported by the findings presented in the article?

Yes

Is the rationale for developing the new software tool clearly explained?

Yes

Is the description of the software tool technically sound?

Partly

Are sufficient details of the code, methods and analysis (if applicable) provided to allow replication of the software development and its use by others?

Yes

Is sufficient information provided to allow interpretation of the expected output datasets and any results generated using the tool?

Yes

Reviewer Expertise:

Metabarcoding ; molecular ecology ; systematics ; mycology

I confirm that I have read this submission and believe that I have an appropriate level of expertise to confirm that it is of an acceptable scientific standard.

# Peer review: Reviewer response for version 1

**DOI:** 10.5256/f1000research.23273.r72941

**Published:** 2020-10-30

**Authors:** Weihong Qi

**Affiliations:** 1 Functional Genomics Center Zurich, Zürich, Switzerland

The authors developed falco, an emulation of the popular FastQC tool, which is faster and can handle very long Nanopore reads. It is a useful development, especially for core facilities and research labs that produce high volumes of sequencing data regularly, where generating read QC reports in a timely fashion is indeed helpful. I only have a few questions and one minor comment:

Main questions:

1. The implementation session could be expanded with more details. From my understanding, the major improvement was identified duplicated analysis in FastQC analysis modules, and implemented a single analysis workflow that was sufficient to generate the same modularized results. But it is not clear to me which changes make falco to handle long ONT reads successfully, while the original FastQC failed.
2. The original FastQC is portable (Unix. Mac and Windows). It also has a GUI version for less experienced users. These features are not important for experienced users and automated workflows where analyzing large amounts of data in a short time is the focus. But they can be important for other type of end users. The authors should at least point out these differences.
3. In results, run times of multiple QC tools analyzing different datasets were compared, how about the RAM usages?

Minor comment:

 The sentence “While FastQC is capable of making summaries for protocols such as 454 ^27^ PacBio ^28^, which generate sequences with around 10,000 bases per read ” should be updated to "While FastQC is capable of making summaries for protocols such as 454 ^27^ PacBio ^28^, which generate sequences with around 10,000-20,000 bases per read".

Are the conclusions about the tool and its performance adequately supported by the findings presented in the article?

Yes

Is the rationale for developing the new software tool clearly explained?

Yes

Is the description of the software tool technically sound?

Yes

Are sufficient details of the code, methods and analysis (if applicable) provided to allow replication of the software development and its use by others?

Yes

Is sufficient information provided to allow interpretation of the expected output datasets and any results generated using the tool?

Yes

Reviewer Expertise:

Genome informatics.

I confirm that I have read this submission and believe that I have an appropriate level of expertise to confirm that it is of an acceptable scientific standard.

# Response

**Published:** 2021-01-09

**Authors:** Guilherme de Sena Brandine

**Affiliations:** University of Southern California, USA

The reviewer has raised some important questions about points not addressed by the manuscript. We provide our responses below, and highlight changes made to the manuscript to address the reviewer’s comments.

*The authors developed falco, an emulation of the popular FastQC tool, which is faster and can handle very long Nanopore reads. It is a useful development, especially for core facilities and research labs that produce high volumes of sequencing data regularly, where generating read QC reports in a timely fashion is indeed helpful. I only have a few questions and one minor comment:*

**

*Main questions:*

*1) The implementation session could be expanded with more details. From my understanding, the major improvement was identified duplicated analysis in FastQC analysis modules, and implemented a single analysis workflow that was sufficient to generate the same modularized results. But it is not clear to me which changes make falco to handle long ONT reads successfully, while the original FastQC failed.*

 We really appreciate the reviewer highlighting the missing details regarding FastQC’s behavior. Upon trying to address this comment, we have further explored the FastQC code to understand why it failed for long reads, and we have learned that the perl script that wraps the FastQC call imposes a maximum memory limit of 250 MB per thread, which we were not aware of at the time we wrote the manuscript, and was prohibitive for the long read samples we have selected. Changing this configuration internally allowed us to run FastQC in the samples that we were previously unable to, thus allowing us to report a more comprehensive comparison of both time and memory for every test we have gathered.  For this reason, we have removed the section named “Falco scales for larger nanopore reads”, instead replacing it with a paragraph at the end of the subsection named “Falco is faster than popular QC tools”, where the memory usage of each mapper is discussed. We have also filled Tables 3 and 4 with the results of running FastQC in the three long-read samples in our tests under the same hardware settings used for other tests.

*2) The original FastQC is portable (Unix. Mac and Windows). It also has a GUI version for less experienced users. These features are not important for experienced users and automated workflows where analyzing large amounts of data in a short time is the focus. But they can be important for other type of end users. The authors should at least point out these differences.*

 We thank the reviewer for this observation. We have made modifications to the first paragraph of the “implementation choices” subsection under “methods” to clarify that falco was designed for UNIX systems and does not include a graphical user interface.

*3) In results, run times of multiple QC tools analyzing different datasets were compared, how about the RAM usages?*

 We appreciate the observation about RAM comparison. We have added two paragraphs in the manuscript that address memory requirement in more detail. A “system requirement” subsection under “methods” was added to emphasize that falco requires about 100 MB for short read samples and under 1 GB for samples with read lengths of at most 1 million. As stated in question (1), we also reported the RAM usage for the software tools compared in the tests, both for short-read and long-read tests in the section “Falco is faster than popular QC tools”.

*Minor comment:*

*The sentence “While FastQC is capable of making summaries for protocols such as 454* *^27^* *PacBio* *^28^* *, which generate sequences with around 10,000 bases per read ” should be updated to "While FastQC is capable of making summaries for protocols such as 454* *^27^* *PacBio* *^28^* *, which generate sequences with around 10,000-20,000 bases per read".*

 We thank the reviewer for the observation. The section containing this sentence was removed given the shift in the focus of the manuscript to memory comparison, as addressed in questions (1) and (3).

# Peer review: Reviewer response for version 1

**DOI:** 10.5256/f1000research.23273.r66327

**Published:** 2020-07-07

**Authors:** R. Henrik Nilsson

**Affiliations:** 1 Gothenburg Global Biodiversity Centre, University of Gothenburg, Gothenburg, Sweden

The authors present a welcome addition to the flora of FastQC-style read processing packages. The fact that it is a drop-in replacement for FastQC is particularly nice.

 The manuscript is a bit too short in my opinion. I miss some background information and some performance-related data. If the authors want to address a wide audience, they should probably work a bit more on the installation instructions and documentation too. The authors should probably also consider defining who their target audience is.

**Introduction:**

- As a discretionary comment: the authors sometimes, but not always, use the Oxford comma (see example below). I wonder if this is something that should be streamlined. “sequencing  data is growing in abundance, dataset size and read length ^9^.” vs. “…adapter  trimming,  filtering  contaminants and low-quality reads, and mapping reads to a reference genome or transcriptome.”
- “as a safety criteria” should probably be “as a safety criterion”.

 

**Methods:**

- Good thinking behind the “We  designed falco ^13^  to  faithfully…” paragraph. Nobody would be helped by yet another set of new file formats. A drop-in replacement is the way to go, if you ask me. And that is indeed what the authors deliver.
- I must make the observation, though, that the name “falco” may not be available (?): https://www.falcoseed.com/ca/article/cibus-registers-new-falco-brand-79k-canola-hybrid/ (some other more or less commercial uses of “Falco” can be found on https://en.wikipedia.org/wiki/Falco). I’m not sure how North American trademark/proprietary laws operate, but I suggest that the authors consult with the lawyers of their university. Better safe than sorry, right. (I consulted with our lawyers at one point, and they had me change the name of a software package we were working on at the time.)
- Two unintended line breaks:
  “uniformly,
  falco’s design centralizes”

 

**Results:**

- Please cite the Sequence Read Archive formally; see Kodama *et al. *(2012 ^1^).
- I like the reproducible nature of the “Results” section.
- Two superfluous line breaks:
  “gzip 
  compressed files.”

 

**Conclusions:**

- Both “Falco” and “falco” are used in the manuscript. You’d think that the name would be fixed as either “Falco” or “falco”.

 

**Software availability:**

- Why not use the term “open source” at least once in the manuscript?

 

**Miscellaneous questions and observations:**

- Out of curiosity: how does falco compare to Liu *et al.* (2019 ^2^)?
- I’ve seen one software tool for quality-score-based trimming of sequences that actually reads the entire query file into memory, and then started to process it. This works less well as data files continues to grow, obviously. Is it worth pointing out that falco does not do this? What is, in fact, the maximum file size allowed by falco? Or is this dictated solely by the operating system?
- You can produce some pretty funny behavior in some other tools for sequence QC/trimming by feeding them a file with a single FastQC entry in it, speaking of nothing. Is there, then, a minimum file size or number of query sequences for falco?
- Unless I’m mistaken, there are no fastq files available on https://github.com/smithlabcode/falco. I think the authors should make one or two available, so that it will be smooth and easy for the reader to try the software out. “The  scripts  used  to  download  files  and  reproduce  the  bench-marking steps described are also available in the same repository within the “benchmark” directory.” comes across as somewhat indirect to me.
- What, exactly, are the hardware and software requirements for falco? This may be worth pointing out. Between the lines I read “any computer you can install the GNU GCC compiler on” – but not all readers will probably read it this way. Instead you’ll get the question: “Does it run on Windows 10?”.
- Also, how much memory is needed to run falco? And how much memory is used up by falco when it processes a large file?
- Between the lines (“Programs were instructed to run using a single thread.”), I take it that falco can use multiple threads. Is that correct? And if so, why not point it out more explicitly? And out of curiosity: when you run falco on 4 cores, do you see a 4x speedup? Or is the bottleneck something else (disk IO?) than raw computational power? How does it scale with the number of cores, in other words?
- Suppose my dataset is 10 Gb, and that I have 15 Gb left of free disk space. Do I dare to run falco on that dataset? How large is the output compared to the input? In a “minimum” (no extra features) as well as “maximum” (all extra features) mode?
- Suppose a user finds a bug, or wants to put forward a feature request. How can the user do that? Should this be mentioned in the manuscript?
- The first question I always get from users of my software tools is: “where can I download the Windows binaries?”. Is it, actually, not a good idea to be explicit about the fact that this is a command-line tool that you compile on your own computer? Would probably save the authors some time to be upfront with this.
- The list of references comes across as somewhat untidy. Some examples follow below. The authors should probably go through all references to make sure they comply with journal specifications.
- () Journal names are sometimes abbreviated, sometimes not. Ref 2 is abbreviated, whereas ref 3 should be abbreviated “BMC Bioinform.”
- () Article titles: should verbs and key nouns in article titles have a leading uppercase letter, as in, e.g., ref 5, or should they not, as in ref 1?
- () Should page ranges be written out in full (ref 23, “1202–1214.”) or should they be abbreviated (ref 25, “1213–8.”)?

 

 Are the installation instructions a bit too thin? I’d say yes, at least if the intention of the authors is to address a diverse audience and not just readers with Linux-style experience. To simulate a less experienced user, I used my son’s MacBook Pro and tried to install falco on it following the manual:

 “Upon downloading, inflating and moving to the source

 directory, installation can be done through the following commands:

 …

 $ ./configure CXXFLAGS="-O3 -Wall"

 $ make all

 $ make install”

 So I did:

 $ cd src

 $ ./configure CXXFLAGS="-O3 -Wall"

 -bash: ./configure: No such file or directory

 And then

 conda install -c bioconda falco

 -bash: conda: command not found

 And that was it. No further clues or assistance to be found in the instructions.

 If the authors are happy with this behavior, then they should make it clear in the manuscript that falco is not for everyone, but rather only for those with significant Linux-style experience.

 “Source code for falco available at: https://github.com/smithlabcode/falco.” – the trailing “.” should be removed, I’d say. The link won’t work for users who copy-and-paste it into their browser. The same thing goes for

 “Archived source code at time of publication: http://doi.org/10.5281/zenodo.3520933 ^13^.” where both the reference and the “.” cause problems.

Are the conclusions about the tool and its performance adequately supported by the findings presented in the article?

Yes

Is the rationale for developing the new software tool clearly explained?

Yes

Is the description of the software tool technically sound?

Partly

Are sufficient details of the code, methods and analysis (if applicable) provided to allow replication of the software development and its use by others?

Yes

Is sufficient information provided to allow interpretation of the expected output datasets and any results generated using the tool?

Yes

Reviewer Expertise:

Metabarcoding ; molecular ecology ; systematics ; mycology

I confirm that I have read this submission and believe that I have an appropriate level of expertise to confirm that it is of an acceptable scientific standard, however I have significant reservations, as outlined above.

## References

[1] : The Sequence Read Archive: explosive growth of sequencing data. *Nucleic Acids Res*.2012;40(Database issue) : 10.1093/nar/gkr854 D54-6 10.1093/nar/gkr854 22009675PMC3245110

[2] : FastProNGS: fast preprocessing of next-generation sequencing reads. *BMC Bioinformatics*.2019;20(1) : 10.1186/s12859-019-2936-9 345 10.1186/s12859-019-2936-9 31208325PMC6580563

# Response

**Published:** 2021-01-09

**Authors:** Guilherme de Sena Brandine

**Affiliations:** University of Southern California, USA

The reviewer has presented a thorough feedback to the description of the Falco software tool, as well as its implementation, description and documentation. We truly appreciate the very helpful comments, and provide our responses to improvement suggestions below. Comments were divided and numbered to allow us to refer to them in other places in our response if certain modifications to the manuscript pertain to multiple comments.

*(1) The authors present a welcome addition to the flora of FastQC-style read processing packages. The fact that it is a drop-in replacement for FastQC is particularly nice.*

**

*The manuscript is a bit too short in my opinion. I miss some background information and some performance-related data. If the authors want to address a wide audience, they should probably work a bit more on the installation instructions and documentation too. The authors should probably also consider defining who their target audience is.*

 We appreciate the comments on the introduction. We have expanded on the target audience on further comments (we address these in more detail on comment 16). We also fully agree that more background information can be provided. We expanded the second paragraph of the “introduction” section to add a brief description of some common quality control tests applied to most next-generation sequencing datasets.

*(2) As a discretionary comment: the authors sometimes, but not always, use the Oxford comma (see example below). I wonder if this is something that should be streamlined. “sequencing data is growing in abundance, dataset size and read length ^9^.” vs. “…adapter trimming, filtering contaminants and low-quality reads, and mapping reads to a reference genome or transcriptome.”*

 We thank the reviewer for this observation. We have revised the manuscript and ensured that the Oxford comma is adopted across the entire manuscript.

*(3) “as a safety criteria” should probably be “as a safety criterion”.*

 We fully agree with the reviewer. This correction was made on the manuscript.

*(4) Good thinking behind the “We designed falco ^13^ to faithfully…” paragraph. Nobody would be helped by yet another set of new file formats. A drop-in replacement is the way to go, if you ask me. And that is indeed what the authors deliver.*

 We appreciate the comments, thank you!

*(5) I must make the observation, though, that the name “falco” may not be available (?):* *https://www.falcoseed.com/ca/article/cibus-registers-new-falco-brand-79k-canola-hybrid/* *(some other more or less commercial uses of “Falco” can be found on https://en.wikipedia.org/wiki/Falco). I’m not sure how North American trademark/proprietary laws operate, but I suggest that the authors consult with the lawyers of their university. Better safe than sorry, right. (I consulted with our lawyers at one point, and they had me change the name of a software package we were working on at the time.)*

 We really appreciate the reviewer raising the possible legal issue that may arise from the program name. We have researched the matter and believe that the software name should not raise legal issues, both due to the program not having any commercial or profitable goals and the name “Falco” being a common proper noun in many Latin languages.

*(6) Two unintended line breaks: “uniformly, falco’s design centralizes”*

 We thank the reviewer for this observation. This line was removed from the manuscript.

*(7) Please cite the Sequence Read Archive formally; see Kodama et al. (2012* *^1^* *).*

 We followed the reviewer’s suggestion and added the appropriate citation when referring to the Sequence Read Archive. We also corrected the meaning of the acronym from "Sequencing Read Archive", as it was previously written.

*(8) I like the reproducible nature of the “Results” section.*

 We appreciate the comment, thank you!**

*(9) Two superfluous line breaks: “gzip compressed files.”*

 We have removed the line break from the manuscript.

*(10) Both “Falco” and “falco” are used in the manuscript. You’d think that the name would be fixed as either “Falco” or “falco”.*

 We thank the reviewer for this observation. The notation used in the manuscript used “Falco” at the start of sentences and “falco” everywhere else, to resemble the name of the binary program used in the command-line interface. We have modified the manuscript to use “Falco” everywhere except in the “use cases” section, where an example command-line call for the program is shown.

*(11) Why not use the term “open source” at least once in the manuscript?*

 We thank the reviewer for this observation. To address this, we started the “Implementation choices” subsections with the following sentence: *Falco* *is an Open Source C++ implementation of the* *FastQC* *software tool built for UNIX-based operating systems.*

*(12) Out of curiosity: how does falco compare to Liu et al. (2019* *^2^* *)?*

 We have downloaded the software from the URL provided in the manuscript (github.com/megagenomics/fastprongs) and performed comparisons on identical hardware to what was used in the manuscript. We tested on two datasets: Dataset 1 consisted of 76 million 150 base reads from arabidopsis (SRR12075121 in SRA), and dataset 2 contained 139 million 100 base reads from chicken (SRR5015166 in SRA). On dataset 1, Falco ran in 9:40 and FastProNGS ran in 5:16 with 3 threads (the default program configuration) and 10:42 on a single thread. On dataset 2, Falco ran in 14:30 and FastProNGS ran in 7:55 with 3 threads and 16:13 with a single thread. We noticed that FastProNGS only reports the following modules in their output: Basic statistics, adapter content, per base sequence quality, per base sequence content and sequence length distribution. We also tried to run Falco by enabling only these modules. Under these settings Falco ran in 4:35 for dataset 1 and 7:12 for dataset 2. In all tests, Falco ran with 92 MB of RAM, whereas FastProNGS used 1.26 GB. In a long-read dataset (test 12 in the manuscript), FastProNGS did not run successfully unless we configured it to only consider the first 200 bases of each read, in which case FastProNGS ran in 2 seconds, but only reported summaries for the first 200 bases of all reads. Falco ran in 12 seconds for this dataset.

 A very meaningful conclusion of this comparison is the potential advantage of multithreading in QC, as evidenced by the steep decrease in processing time from FastProNGS when multithreading is enabled. We noticed, upon inspecting its source code, that this performance improvement can be explained by FastProNGS reading multiple reads in batch and allowing a new set of reads to be loaded while the previous batch of reads is processed. In contrast, Falco loads and processes each read sequentially, which reduces RAM usage but makes multithreading difficult in its current implementation. The performance of FastProNGS suggests that switching to a “batch processing” paradigm may have significant speed advantages when multiple cores are used and enough RAM is available to load reads in batch, and this is something we will incorporate in future versions of Falco, especially in order to address (18). We thank the reviewer for bringing this tool to our attention.

**

*(13) I’ve seen one software tool for quality-score-based trimming of sequences that actually reads the entire query file into memory, and then started to process it. This works less well as data files continues to grow, obviously. Is it worth pointing out that falco does not do this? What is, in fact, the maximum file size allowed by falco? Or is this dictated solely by the operating system?*

 Both disk and memory requirements will depend on the length of the largest read in the dataset, as Falco processes the input FASTQ one read at a time. To address the computational requirements necessary to run Falco in more detail in the manuscript, we created an additional subsection named “system requirements” under the “Methods” section, where the computational resources (memory and disk) required to run Falco successfully are discussed. Furthermore, we have added a paragraph in the “results” section summarizing the memory and disk usage for the tests used for comparison across programs.

**

*(14) You can produce some pretty funny behavior in some other tools for sequence QC/trimming by feeding them a file with a single FastQC entry in it, speaking of nothing. Is there, then, a minimum file size or number of query sequences for falco?*

 There are no minimum or maximum file sizes required by Falco. We have tested (although not disclosed in the manuscript) that Falco successfully runs on empty files and single-read files. We thank the reviewer for having raised this issue, and have addressed that there are no constraints in file size or number of reads in the “system requirements” section stated in (13).

*(15) Unless I’m mistaken, there are no fastq files available on* *https://github.com/smithlabcode/falco* *. I think the authors should make one or two available, so that it will be smooth and easy for the reader to try the software out. “The scripts used to download files and reproduce the benchmarking steps described are also available in the same repository within the “benchmark” directory.” comes across as somewhat indirect to me.*

 We thank the reviewer for this observation. While we cannot provide the full FASTQ files used to perform our comparisons in the GitHub repository, we do agree that the documentation of our tests can be made simpler for users who wish to test the program. We made modifications in our repository to simplify both testing in an example file and testing in the FASTQ files used in the manuscript for comparison. Specifically, we added (1) direct links to the SRA files under the “benchmark” directory and (2) an “example.fq” file, consisting of a FASTQ file of 1000 reads, which is used as input for the example commands provided in the README.**

*(16) What, exactly, are the hardware and software requirements for falco? This may be worth pointing out. Between the lines I read “any computer you can install the GNU GCC compiler on” – but not all readers will probably read it this way. Instead, you’ll get the question: “Does it run on Windows 10?”.*

 We agree that constraints should be disclosed in more detail, and that the limited support for usage of Falco on Windows should be more explicit. We have rephrased the first paragraph in the “implementation choices”. The last sentences disclose more explicitly that Falco, by design, is a UNIX-centric program made to be run on a command line and that, unlike FastQC, it cannot be run in a graphical user interface.

*(17) Also, how much memory is needed to run falco? And how much memory is used up by falco when it processes a large file?*

 Falco requires under 1 GB of memory for any short or long read file generated by the current sequencing technologies. More memory will be required when technologies expand read lengths to the order of millions or billions of bases per read. We have added a discussion of disk and memory requirements under the “systems requirement” section, and also discussed the memory usage of the programs compared in the manuscript under the section “Falco is faster than popular QC tools”.

*(18) Between the lines (“Programs were instructed to run using a single thread.”), I take it that falco can use multiple threads. Is that correct? And if so, why not point it out more explicitly? And out of curiosity: when you run falco on 4 cores, do you see a 4x speedup? Or is the bottleneck something else (disk IO?) than raw computational power? How does it scale with the number of cores, in other words?*

 Falco, like FastQC currently does not use multiple threads to process a single file, and no significant speed difference was observed when running fastp with multiple threads, which is why we focused our comparison on single-thread across the software tools. Despite QC computations being fast relative to IO, our comparison with FastProNGS described in (12) suggests that multithreading can lead to speed improvements if reading and processing are done in parallel, and we certainly plan on exploring this paradigm in the next release of Falco. We have also rephrased the third paragraph of the subsection “Falco is faster than popular QC tools” to say “Both fastp and fastqc were instructed to run on a single thread” to avoid ambiguities regarding Falco’s multithread option.

*(19) Suppose my dataset is 10 Gb, and that I have 15 Gb left of free disk space. Do I dare to run falco on that dataset? How large is the output compared to the input? In a “minimum” (no extra features) as well as “maximum” (all extra features) mode?*

 We have addressed the disk requirement on the “system requirements” section disclosed in (13) and (14), specifically adding the sentence “The total disk space required to store the three output files generated by Falco is under 1 MB”. Like FastQC, Falco’s output is a set of reports whose size scales with the maximum read length of the input but are never under 1 MB in total. We fully agree that the fact that disk space is not crucial to run Falco should be made more explicit.

*(20) Suppose a user finds a bug, or wants to put forward a feature request. How can the user do that? Should this be mentioned in the manuscript?*

 We thank the reviewer for this observation. All issues and bug reports can be done through our GitHub page, the same one provided for the source code. To make this clearer for users, we have added a sentence at the “software availability” section, stating that errors, installation problems and bugs can be reported in the “Issues” section in the same URL provided to download the source code.

*(21) The first question I always get from users of my software tools is: “where can I download the Windows binaries?”. Is it, actually, not a good idea to be explicit about the fact that this is a command-line tool that you compile on your own computer? Would probably save the authors some time to be upfront with this.*

 We fully agree with the reviewer, and have added the statements of a more specific target audience as discussed in (16).

*(22) The list of references comes across as somewhat untidy. Some examples follow below. The authors should probably go through all references to make sure they comply with journal specifications.*

*Journal names are sometimes abbreviated, sometimes not. Ref 2 is abbreviated, whereas ref 3 should be abbreviated “BMC Bioinform.”*

*Article titles: should verbs and key nouns in article titles have a leading uppercase letter, as in, e.g., ref 5, or should they not, as in ref 1?*

*Should page ranges be written out in full (ref 23, “1202–1214.”) or should they be abbreviated (ref 25, “1213–8.”)?*

 We really appreciate the keen observations on the reference standards. We have reviewed our citations and standardized journals to their non-abbreviated names, uppercase letters only in leading words, and pages written in full.

*(26) Are the installation instructions a bit too thin? I’d say yes, at least if the intention of the authors is to address a diverse audience and not just readers with Linux-style experience. To simulate a less experienced user, I used my son’s MacBook Pro and tried to install falco on it following the manual:*

**

*“Upon downloading, inflating and moving to the source*

*directory, installation can be done through the following commands:*

*…*

*$ ./configure CXXFLAGS="-O3 -Wall"*

*$ make all*

*$ make install”*

**

*So I did:*

**

*$ cd src*

*$ ./configure CXXFLAGS="-O3 -Wall"*

*-bash: ./configure: No such file or directory*

**

*And then*

**

*conda install -c bioconda falco*

*-bash: conda: command not found*

**

*And that was it. No further clues or assistance to be found in the instructions.*

**

*If the authors are happy with this behavior, then they should make it clear in the manuscript that falco is not for everyone, but rather only for those with significant Linux-style experience.*

 We really appreciate the reviewer bringing up this **observation about our documentation. We agree that the wording of “moving to the source directory” was misleading and may cause users to try to run the commands on the “src” directory. We have updated our README with clearer command line instructions that show the user how to clone the repository or download a release file, as well as which directory to move to in order to run the commands. We are striving to make the documentation as clear and simple as possible, and truly appreciate these suggestions on how these can be improved.

**

*(27) “Source code for falco available at:* *https://github.com/smithlabcode/falco* *.” – the trailing “.” should be removed, I’d say. The link won’t work for users who copy-and-paste it into their browser. The same thing goes for*

*“Archived source code at time of publication: http://doi.org/10.5281/zenodo.3520933 ^13^.” where both the reference and the “.” cause problems.*

 We appreciate this observation and the potential problems punctuation near links may cause. We have ensured that the trailing dots and citations are clearly separated from the links and that they will not cause problems when copying URLs directly from the manuscript.
